# Surgical Management of Pulmonary Typical Carcinoids: A Single-Centre Experience Comparing Anatomical and Non-Anatomical Resections

**DOI:** 10.3390/jcm14155488

**Published:** 2025-08-04

**Authors:** Carmelina Cristina Zirafa, Beatrice Manfredini, Gaetano Romano, Ilaria Ceccarelli, Fabrizia Calabrò, Riccardo Morganti, Greta Alì, Franca Melfi, Federico Davini

**Affiliations:** 1 Minimally Invasive and Robotic Thoracic Surgery, Department of Surgical, Medical, Molecular and Critical Care Pathology, University Hospital of Pisa, Via Paradisa 2, 56124 Pisa, Italy; carmelina.zirafa@ao-pisa.toscana.it (C.C.Z.); gaetano.romano@ao-pisa.toscana.it (G.R.); f.davini@ao-pisa.toscana.it (F.D.); 2 Division of Thoracic Surgery, Department of Surgical Medical and Molecular Pathology and Critical Area, University Hospital of Pisa, 56124 Pisa, Italy; i.ceccarelli1@studenti.unipi.it (I.C.); fabrizia.calabro@phd.unipi.it (F.C.); 3 Section of Statistics, University Hospital of Pisa, 56124 Pisa, Italy; r.morganti@ao-pisa.toscana.it; 4 Pathological Anatomy, Department of Surgical, Medical, Molecular and Critical Care Pathology, University Hospital of Pisa, 56124 Pisa, Italy; g.ali@ao-pisa.toscana.it; 5 Unit of Thoracic Surgery, Department of Pharmacy and Health and Nutrition Sciences, University of Calabria, 87036 Rende, Italy; franca.melfi@unical.it

**Keywords:** typical carcinoid, lung neuroendocrine tumor, anatomical resection, non-anatomical resection, survival

## Abstract

**Background/Objectives:** Pulmonary typical carcinoid (TC) is a rare type of primary neuroendocrine neoplasm of the lung with indolent behavior and a good prognosis. The main treatment strategy is surgery, the extent of which is controversial given the nature of the disease. The aim of this study is to assess whether the extent of resection influences survival and recurrence in patients undergoing lung resection and lymphadenectomy for TC and to investigate negative prognostic factors for OS. **Methods**: A single-centre retrospective study of 15 years’ experience was conducted. Data from all patients who underwent lung resection and lymphadenectomy for TC were collected. Patients were divided into two groups: anatomical and non-anatomical resections. Perioperative and long-term oncological results were analyzed. **Results**: In total, 115 patients were surgically treated for TC, of whom 83 (72%) underwent anatomical resection and 32 (28%) non-anatomical resection. Univariate analyses showed that age, left lower lobe, and many comorbidities had a detrimental effect on OS, whereas on multivariate analysis, only left lower lobe location and a high Charlson–Deyo comorbidity index (CCI) were confirmed as negative prognostic factors for OS. At a median follow-up of 93 months (IQR 57-129), the OS survival curves show a slightly lower trend for non-anatomical resections (*p* 0.152), while no differences were found for DFS. **Conclusions**: The results of this study confirm that in selected patients at risk for major resections, non-anatomical resection can be used to treat TC when R0 is achievable. These data, together with evidence from the literature, highlight the importance of patient-centred care in this rare disease.

## 1. Introduction

Lung neuroendocrine tumors (L-NETs) account for 20% of primary lung cancers, whereas carcinoids represent 2% (with a typical to atypical ratio of 10:1) [1].

In the latest World Health Organization (WHO) 2021 classification, L-NETs comprise four subtypes that differ significantly in their biological behavior. Typical carcinoids (TCs) and atypical carcinoids (ACs) are well-differentiated, low-grade and intermediate-grade tumors, respectively, whereas large cell neuroendocrine carcinoma (LCNEC) and small cell lung cancer (SCLC) are poorly differentiated, high-grade tumors [1]. The distinguishing diagnostic criteria for well-differentiated L-NETs are defined by two main factors: firstly, the mitotic count, and secondly, the presence and extent of necrosis. TC is characterized by a mitotic count of less than 2 per 10 high-power fields (HPF) and the absence of necrosis, whereas AC is defined by a mitotic count of at least two 2 per 10 HPF and/or the presence of necrosis [1,2,3].

The incidence of lung carcinoid is extremely low, ranging from 0.2 to 2/100,000 per year in both the USA and Europe. There has been an increase in the number of cases, which is probably due to greater awareness and improved diagnostic techniques [4,5].

TC is regarded as an indolent tumor, characterized by low recurrence rates and a favorable prognosis. Surgery is preferred treatment option, with 5- and 10-year survival rates exceeding 90% [6,7]. Notwithstanding the aforementioned characteristics, anatomical resection, accompanied by the dissection of mediastinal lymph nodes, is currently recognised as the gold standard treatment for stages I-IIIA carcinoids, in accordance with the most recent National Comprehensive Cancer Network (NCCN) and European Society of Medical Oncology (ESMO) guidelines [8,9].

Recently, a significant number of clinicians have expressed skepticism regarding the potential for the treatment of these neoplasms with lung parenchymal-sparing resections, given their tendency to exhibit a slow growth pattern. Retrospective studies have shown that sublobar resections are non-inferior to lobar resections in terms of survival [10,11,12,13,14,15,16]. Nevertheless, there has been an absence of sufficient evidence to promote this as a standard of care.

It is evident that, to date, the 2015 European Neuroendocrine Tumor Society Expert Consensus Guidelines still confirm anatomical complete resection and systematic lymphadenectomy as the preferred treatment modality [17,18,19,20], with sublobar resection being designated for patients considered to be at high-risk. In the most recent International Association for the Study of Lung Cancer (IASCL) best practice guidelines, produced by a collaboration between the Commonwealth Neuroendocrine Tumour Research Collaboration (CommNETs) and the North American Neuroendocrine Tumour Society (NANETS) [21], the statements on surgery have been revised to update the level of evidence, supporting sublobar resection for peripheral TC < 2 cm, and to reflect the growing body of evidence [10,11,12,13,14,15].

The optimal therapeutic approach for the typical carcinoid tumour is currently under discussion, and, given the non-aggressive nature of the typical carcinoid, the potential role of non-anatomical resection remains to be defined.

The objective of this study is to make a comparison between non-anatomical and anatomical resection in the treatment of TC, in order to evaluate the survival and recurrence rates, both in the overall cohort and in the subgroup of stage I patients. Furthermore, the study sought to identify negative prognostic factors for survival in order to improve the management of patients with this rare malignancy.

## 2. Materials and Methods

A single-centre retrospective study was conducted, with data collected on all patients who underwent surgery for TC between 2008 and 2022. The inclusion criteria encompassed the following factors: a preoperative computed tomography (CT) scan, TC histology, surgery for the complete removal of neoplasm(R0), lymphadenectomy, and the availability of oncological follow-up data.

Patients were divided into two groups according to the type of surgery performed:

- Non-anatomical resection, including segmentectomies performed for tumors greater than 2 cm in diameter, wedge resections, and bronchotomy with bronchoplasty (with exclusive resection of the bronchial tissue);
- Anatomical resection, including segmentectomy for tumors less than or equal to 2 cm in diameter, lobectomy, pneumonectomy, and sleeve lobectomy.

Patients undergoing segmentectomy for tumours larger than 2 cm could not undergo major resections due to the functional characteristics and could not undergo wedge resections due to the location of the neoplasm. The following surgical approaches employed at our centre were included: thoracotomic, videothoracoscopic, and robotic surgery.

Furthermore, the patients were further subdivided according to the location of the tumor, as follows:

- Peripheral (in the outer third of the lung);
- Central;
- Endo-peribronchial, defined as any tumour visualized directly during bronchoscopy or in association with lung atelectasis and/or obstructive pneumonia [22] or close to the nearest bronchus at a CT scan.

The preoperative cytohistological diagnosis of the pulmonary lesion was not considered in the present analysis, as patients with a definite diagnosis of typical carcinoid represented an insufficient sample size for statistical evaluation.

Histological details, such as mitotic index and Ki67 expression rate, were also recorded. Demographic, clinical, and pathological characteristics and perioperative outcomes were systematically collected and analyzed.

The assessment of comorbidity was conducted utilizing the Charlson–Deyo comorbidity index (CCI) [23].

Prior to undergoing surgery, patients were subjected to respiratory function assessment, cardiological evaluation, blood tests, and anesthetic assessment. Post-operative complications were collected according to the Clavien–Dindo Complications Classification (CDCC) [24]. The term “Perioperative mortality” is defined as any death occurring within the first 30 days of surgery.

The histological diagnosis of TC was based on the WHO 2021 classification of lung tumors [1] and Travis’ histological guidelines for the diagnosis of L-NETs [25]. The pathological stage was determined in accordance with the 8th edition of the TNM Staging of Lung Cancer [26].

All patients underwent clinical–radiological follow-up. In the event of any radiological suspicion of recurrence, the patient underwent 68Ga-DOTATOC PET-CT, CT-guided biopsy, or bronchoscopy-guided biopsy, as determined by the attending medical professionals.

The patient survival data were analyzed in terms of overall survival (OS), cancer-specific survival (CSS), and disease-free survival (DFS), in the entire cohort of patients and then only in stage I patients. OS is defined as the time from diagnosis of the disease to death from any cause. CSS is defined as the time from diagnosis to death from lung cancer. DFS is defined as the time from diagnosis to any recurrence or death.

### Statistics

Categorical data were described with absolute and relative frequency (%); continuous data were summarized with mean and standard deviation. Survival curves were calculated using the Kaplan–Meier method and the log-rank test was applied to evaluate differences between curves. Analysis of the OS influencing factors was performed by univariate and multivariate (step-wise) Cox regression. The results of the Cox regression were expressed by HR and 95% CI. Significance was set at 0.05 and all analyses were carried out with SPSS v.29 technology.

## 3. Results

From January 2008 to December 2022, 115 patients underwent lung resection with mediastinal lymph node dissection or sampling for the treatment of TC. Of these patients, 102 underwent stage I treatment (Table 1).

The majority of these lesions were found to be endobronchial (70%) and central (56%). Table 1 summarizes the characteristics of the whole and stage I populations.

Concerning the type of resection, 83 anatomical resections were performed, including 67 lobectomies, of which 3 were combined with bronchoplasty, 2 bilobectomies, 1 pneumonectomy, 11 segmentectomies, and 2 sleeve lobectomies. Furthermore, 32 non-anatomical resections were performed: 23 wedge resections, 8 segmentectomies for cancers greater than 2 cm in maximum diameter, and 1 bronchotomy with bronchoplasty

An analysis of the baseline characteristics revealed no statistically significant differences between the two groups.

Perioperative results of the entire population and stage I patients are described in Table 2.

Radical resection (R0) was achieved in all patients.

Post-operative complications occurred in 13 patients (Table 2), all of which were of minor grade according to CDCC (grade I–II): 4 cases of prolonged air leaks, 2 cases of anemia requiring transfusions, 1 case of stroke, 5 cases of atelectasis treated with bronchoscopy, and 3 instances of atrial fibrillation.

The perioperative findings and histopathological features are shown in Table 2.

**Figure 1:**
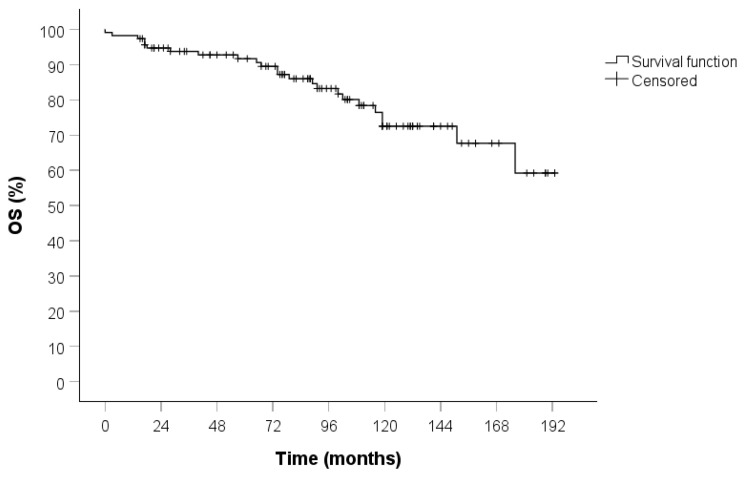
OS curve in the entire cohort calculated by the Kaplan–Meier method (115 patients).

**Figure 2:**
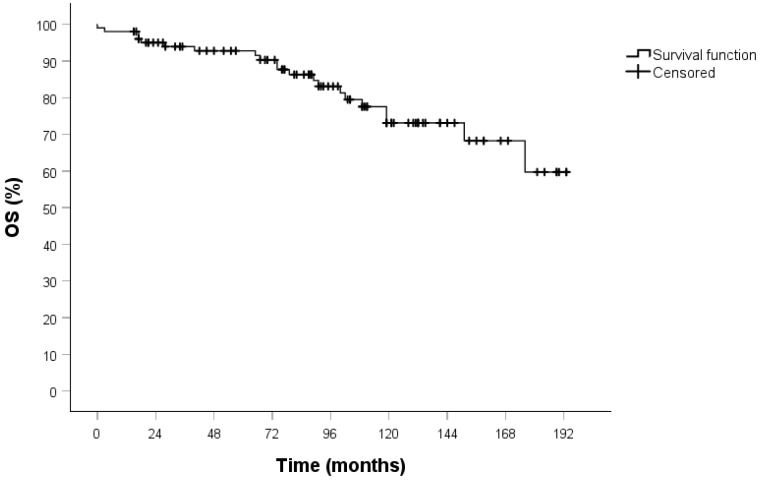
OS curve in the stage I population calculated by the Kaplan–Meier method (102 patients).

All patients underwent clinical and radiological follow-up; the median duration of which was 93 months (IQR 57-129). No patients were lost to follow-up. The 5-year OS in both the overall cohort (Figure 1) and the stage I population (Figure 2) was 94%.

During the follow-up period, four (3.5%) recurrences were observed, all of which occurred in the anatomical resection group. The sites of recurrence were as follows: contralateral lung in two cases, homolateral lymph nodes and bone in one case, and finally liver in one patient. The median time to recurrence was 66 months (range 18–144), with two cases recurring within 24 months and the others at 9 and 12 years, respectively. It is noteworthy that all cases of recurrence had pN0 status at the time of the surgery. In the stage I group, three of these recurrences were observed.

Due to the limited number of cancer-related deaths (n = 2), of which only one occurred in the stage I group, it was not feasible to statistically analyze the Kaplan–Meier curve for CSS. As a secondary observation, we point out that these deaths occurred in the anatomical resection group.

Univariate analysis of the entire cohort demonstrated that older patients with a higher number of comorbidities who had the lesion in the left lower lobe had a poorer survival. Moreover, multivariate analysis confirmed that a higher CCI index and left lower lobe were negative prognostic factors for OS (Table 3). The other factors had no impact on OS at univariate analysis, i.e., type of resection, pStage, pT, tumor size, other tumor localizations besides the left lower lobe, number of mitoses, as well as other patient-related and perioperative characteristics, as can be seen in Table 3. The impact of pathological lymph node status was not evaluated in this study, as no patients with pN2 were included in this study and N1 metastases were found in only four patients.

Univariate analysis was also performed in the stage I population, which showed that only more comorbidities, as assessed by the CCI index, negatively influenced OS, a finding that was confirmed on multivariate analysis (Table 4).

**Figure 3:**
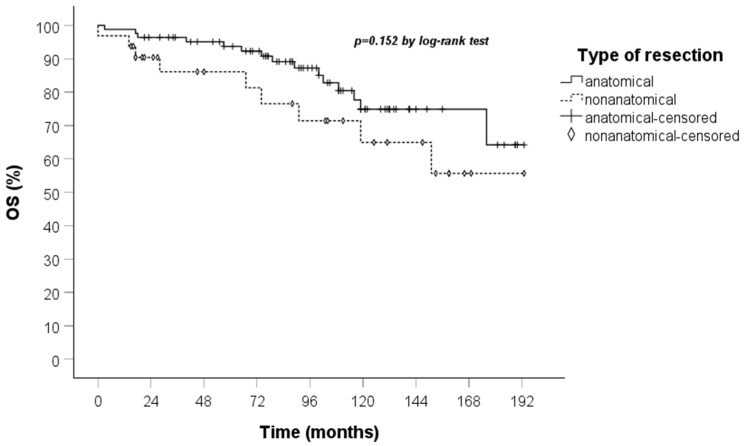
OS curve calculated by the Kaplan–Meier method according to the type of resection (83 patients anatomical vs. 32 non-anatomical).

**Figure 4:**
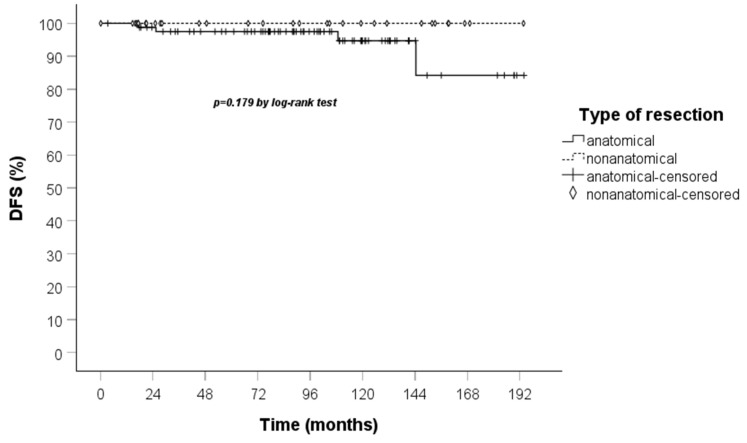
DFS curve calculated by the Kaplan–Meier method according to the type of resection (83 patients anatomical vs. 32 non-anatomical).

A comparison of the Kaplan–Meier curves for OS and DFS for the anatomical and non-anatomical resection groups was conducted. The results indicated that non-anatomical resections showed a marginally lower OS, though this did not reach statistical significance, in the treatment of TC when compared to anatomical resections. Furthermore, the DFS curve demonstrated that recurrences occurred exclusively within the anatomical resection group (Figure 3 and Figure 4).

**Figure 5:**
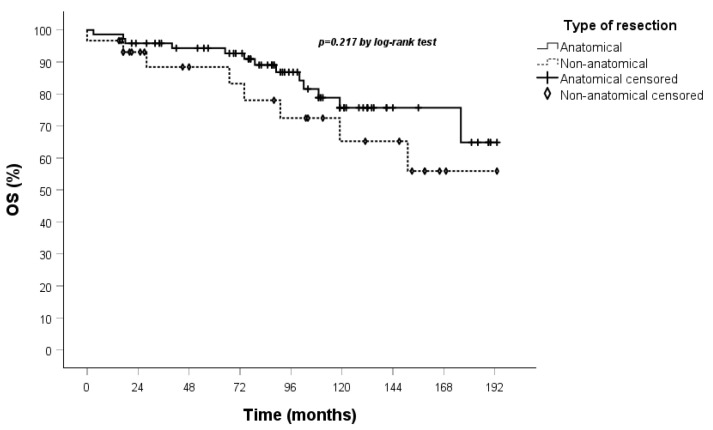
OS curve calculated by the Kaplan–Meier method according to the type of resection (72 patients anatomical vs. 30 non-anatomical).

**Figure 6:**
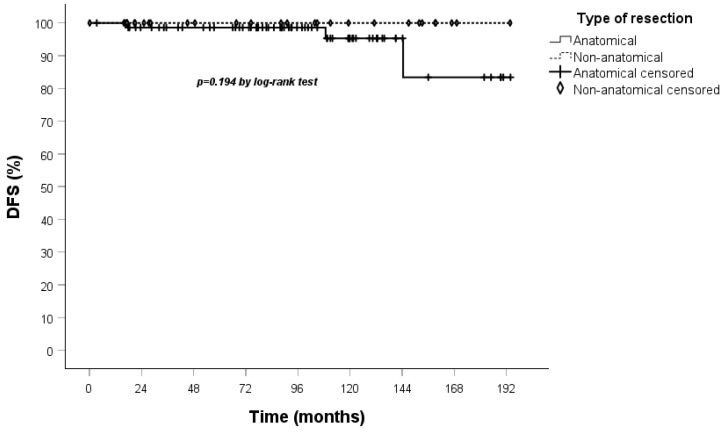
DFS curve calculated by the Kaplan–Meier method according to the type of resection (72 patients anatomical vs. 30 non-anatomical).

These results were confirmed when Kaplan–Meier curves were performed on OS and DFS in the stage I population (Figure 5 and Figure 6).

## 4. Discussion

In 1998, lung typical carcinoids were redefined by Travis et al. as L-NETs with a mitotic count of less than 2 per 2 mm^2^ (10 HPF) without evidence of necrosis [27], in contrast to Argoni’s original classification, which considered neuroendocrine tumors with less than five mitoses per 2 mm^2^ as TC [28,29]. In 1999, the WHO published a new classification of pulmonary carcinoids, adopting the Travis classification and clearly distinguishing TC and AC as a prognostically distinct subset of L-NETs [28].

The most effective treatment for TC is surgery, and the indications for its management are related to those for non-small cell lung cancer (NSCLC) because it is a rare neoplasm, and there are no randomized trials.

Since TC is an indolent neoplasm with a favorable prognosis, several retrospective studies have been conducted over the years to investigate the possibility of treatment with parenchymal sparing resection and to identify possible negative prognostic factors for OS.

A considerable number of retrospective studies in the literature have analyzed pulmonary carcinoids globally [13,30,31,32], without taking into account that AC and TC have different biological aggressiveness, which has an impact on survival outcomes.

Given the difficulty in achieving large samples to obtain significant results, large registry-based studies have been conducted [10,11,12,33,34]. These studies have shown no difference in five-year overall survival rates among patients treated with lobar or sublobar resection for small TC.

Based on these findings, a novel recommendation has been proposed in the recent CommNETs-NANETS guidelines. These recommendations suggest that peripheral TC without lymph node involvement and smaller than 2 cm (stage I) can be managed with R0 sublobar resection [21]. Furthermore, the most recent NCCN guidelines posit that sublobar resection may be as effective as lobectomy if complete resection of the tumor is feasible, as evidenced by retrospective analyses [8].

However, disparate results have been reported by certain studies, which have observed a suboptimal survival rate in patients with typical carcinoids undergoing sublobar resections [17,18]. The patient cohort was typically distinguished by advanced age and more severe clinical conditions, both of which have a negative influence on OS.

In contrast, Yang observed that there were no differences in survival among patients older than 65 years who underwent lobar and sublobar resections, thus confirming the need for research with more homogeneous samples [15].

In the series of patients evaluated in our study, the median age was similar, and no statistically significant difference (*p* 0.152) was observed in terms of OS between the non-anatomical resection and the anatomical resection group.

The findings of the present study demonstrated that patients who underwent non-anatomical resections have a lower recurrence rate in comparison to patients who underwent anatomical resections. Although studies evaluating the impact of the extent of lung resection on survival are varied, these usually do not analyze DFS, so data on recurrences in lung carcinoid patients undergoing surgery are lacking. However, when evaluating the outcomes of surgical resection, it would also be crucial to evaluate the DFS, given the undeniable impact that recurrences have on patients’ quality of life.

Subsequently, retrospective studies have also been conducted in order to assess the difference in survival between segmentectomy and wedge resection in the treatment of typical carcinoids. In 2019, Yan et al. conducted a retrospective study based on data from 1887 TC patients, finding that the prognostic outcome of comparing wedge resection and segmentectomy was not statistically significant [16]. In 2022, Bekcman et al. [14] observed the same oncological result in the treatment of clinical T1N0M0 typical bronchopulmonary carcinoids comparing segmental and wedge resection, confirming non-anatomical resection as a reasonable alternative.

In accordance with these findings, the present study revealed that the type of resection was not a negative prognostic factor for OS in univariate (*p* 0.159) and multivariate (*p* 0.056) analyses, as corroborated by univariate analysis (*p* 0.223) conducted on the stage I population exclusively. These oncological findings, in conjunction with the evidence presented in this report, suggest that patients afflicted with numerous comorbidities and a neoplasm situated in the left lower lobe are likely to experience a more unfavorable prognosis with regard to survival. This could assist surgeons in the process of determining the most appropriate treatment strategy for these rare tumors, namely, identifying cases that are suitable for non-anatomical resection when complete resection is feasible.

Other authors have attempted to identify prognostic factors for OS in the treatment of TC to support clinicians in choosing the type of resection to perform. They describe older age and worse condition as negative prognostic factors [18,35,36]. In 2015, a prognostic model for TC survival based on the European Society of Thoracic Surgeon Neuroendocrine Tumours Working Group (NET-WG) database was presented, identifying age, gender, previous malignancies, peripheral tumor, TNM stage, and ECOG PS as negative predictive variables: in higher score patients, a closer follow-up should be required [36].

Otherwise, the results of the study indicate that pathological stage or tumor dimension did not influence OS in the univariate analysis. In addition, the Kaplan–Meier survival curves for the general cohort, including patients from stage I to stage IIIA and those for stage I patients are overlapping. This result is in contrast with the validations for NSCLC [37], as well as the evidence from the literature on neuroendocrine lung neoplasms, where, in several retrospective studies including both TC and AC, pathological stage influences mortality [13,30,38]. This highlights the necessity to develop specific treatment recommendations for TC, which exhibits markedly different behavior and prognosis from both NSCLC and AC.

Furthermore, systematic lymph node dissection remains recommended in cases of typical carcinoid, although studies have observed a trend toward a lower five-year survival for CT patients with lymph node involvement undergoing chemotherapy after surgery, compared to patients undergoing post-surgical observation [39,40]. As mentioned above, it was not feasible to assess the impact of lymphadenectomy on oncological outcomes in our analyzed sample due to the limited number of patients with nodal involvement.

Further studies are required to clarify the role of lymphadenectomy in relation to the limited benefit of adjuvant therapy and the indolent nature of typical carcinoids.

The limitations of this study are its retrospective and single-centred nature, resulting in a small cohort of patients. A further limitation is the restricted statistical power, attributable to the low frequency of recurrences and deaths that characterize this neoplasm. This is corroborated by the findings of this study, which make it difficult to provide a response regarding the impact of the type of resection on survival.

A significant strength of this study is the extended follow-up period, which is crucial for accurately assessing the impact of the tumor on survival, particularly given the low aggressiveness of the neoplasm. Moreover, the homogeneity of the sample considered in this study corroborates the results obtained.

## 5. Conclusions

The present study has demonstrated that non-anatomical resections for TC result in comparable surgical and long-term oncological outcomes to anatomical resections, with no statistically significant differences found. Furthermore, patients with a high CCI index and left lower lobe neoplasm present a worse prognosis in terms of OS. The available data, when considered in conjunction with the evidence in the literature, suggests that this rare disease should be evaluated by a multidisciplinary tumor board comprising experts in the field of neuroendocrine tumors to establish a patient-centred management process.

In view of the challenges associated with preoperative biopsies in distinguishing between typical and atypical carcinoids, it is recommended that anatomical resection be considered in patients in good condition, with a preference for resections that spare the lung parenchyma, if feasible. This approach obviates the necessity for subsequent reintervention in instances where an atypical carcinoid diagnosis has been made.

In selected patients, wedge resection with lymphadenectomy may be considered if an R0 resection can be achieved.

## Figures and Tables

**Table 1:** Characteristics of the entire population (n = 115) and the stage I population (n = 102). Statistics: frequency (%) or mean (sd).

	Entire Population	Stage I Population
Characteristics	Statistics	Statistics
Age	63 (13)	64 (12)
Gender		
M	31 (27)	29 (28.4)
F	84 (73)	73 (71.6)
CCI	3.6 (1.6)	3.6 (1.6)
BMI	28 (5.1)	28.3 (5)
History of smoking		
No	56 (49)	49 (48)
Ex	41 (36)	38 (37.3)
Yes	18 (16)	15 (14.7)
Side		
Right	67 (58)	62 (60.8)
Left	48 (42)	40 (39.2)
Location of the tumor		
Central	64 (56)	56 (54.9)
Peripheral	51 (44)	46 (45.1)
Endo-peribronchial lesion		
No	35 (30)	30 (29.4)
Yes	80 (70)	72 (70.6)

**Table 2:** Perioperative outcomes of the entire population (n = 115) and the stage I population (n = 102). Statistics: frequency (%) or mean (sd).

	Entire Population	Stage I Population
Characteristics	Statistics	Statistics
Type of resection		
Anatomical	83 (72)	72 (71)
Non-anatomical	32 (28)	30 (29)
Type of surgery		
Open	64 (56)	57 (56)
Robot	45 (39)	39 (38)
Vats	6 (5)	6 (6)
Post-operative complications		
No	102 (89)	87 (85)
Yes	13 (11)	15 (15)
Chest drain duration (days)	3.7 (2.5)	3.8 (2.6)
Hospital stay (days)	5 (1.7)	5 (1.7)
pT stage		
1a	22 (19)	22 (22)
1b	47 (41)	45 (44)
1c	29 (25)	28 (27)
2a	8 (7)	7 (7)
2b	4 (3)	
3	3 (3)	
4	2 (2)	
pN stage		
0	111 (97)	102 (100)
1	4 (3)	
pStage		
IA1	22 (19)	22 (22)
IA2	45 (39)	45 (44)
IA3	28 (24)	28 (27)
IB	7 (6)	7 (7)
IIA	4 (3)	
IIB	7 (6)	
IIIA	2 (2)	
Ki67%	5.4 (3.5)	5.1 (3)
Mitoses	0.91 (0.4)	0.9 (0.4)
Tumor dimension (mm)	20.5 (12.3)	18.1 (7.9)

**Table 3:** Univariate and multivariate analysis of the factors influencing OS in the entire population.

	Univariate Analysis				Multivariate Analysis (Step-Wise Method)			
	HR	95% CI Lower	95% CI Upper	*p*-Value	HR	95% CI Lower	95% CI Upper	*p*-Value
Gender: (0) M, (1) F	0.785	0.325	1896	0.591				
Age	1.044	1.004	1.086	**0.029**				0.555
History of smoking: (0) no, (1) ex, (2) yes	1.061	0.619	1.820	0.829				
BMI	1.028	0.954	1.108	0.465				
Charlons-Deyo Comorbidity Index	1.696	1.303	2.208	**<0.001**	1.753	1.322	2.325	**<0.001**
Side: (0) dx, (1) sx	1.040	0.459	2.357	0.925				
Endo-peribronchial lesion: (0) no, (1) si	0.611	0.271	1.376	0.234				
Location of the tumor: (0) central, (1) peripheral	1.740	0.778	3.891	0.177				
Type of resection: (0) anatomical, (1) nonanatomical	1.822	0.791	4.197	0.159				0.056
Post-operative complications: (0) no, (1) yes	1.742	0.689	4.400	0.241				
Chest drain duration	0.936	0.746	1.174	0.566				
Hospital stay	1.024	0.810	1.295	0.841				
Mitoses	0.944	0.310	2.869	0.919				
Dimension of the tumor	1.010	0.982	1.039	0.480				
T	1.174	0.645	2.138	0.599				
pStage	1.244	0.504	3.070	0.636				
Left lower lobe: (0) no, (1) yes	2.373	1.031	5.461	**0.042**	2.492	1.069	5.807	**0.034**
Left upper lobe: (0) no, (1) yes	0.676	0.200	2.287	0.529				
Right lower lobe: (0) no, (1) yes	0.737	0.251	2.164	0.578				
Right upper lobe: (0) no, (1) yes	1.045	0.311	3.509	0.943				
Middle lobe: (0) no, (1) yes	0.641	0.237	1.729	0.379				

**Table 4:** Univariate and multivariate analysis of the factors influencing OS in the stage I population.

	Univariate Analysis				Multivariate Analysis (Step-Wise Method)			
	HR	95% CI Lower	95% CI Upper	*p*-Value	HR	95% CI Lower	95% CI Upper	*p*-Value
Gender: (0) M, (1) F	1.115	0.408	3.046	0.832				
Age	1.036	0.994	1.081	0.096	0.994	0.943	1.048	0.830
History of smoking: (0) no, (1) ex, (2) yes	1.156	0.652	2.049	0.620				
BMI	1.008	0.929	1.093	0.853				
Charlons-Deyo Comorbidity Index	1.625	1.230	2.148	**<0.001**	1.655	1.199	2.284	**0.002**
Side: (0) dx, (1) sx	1.259	0.527	3.008	0.605				
Endo-peribronchial lesion: (0) no, (1) si	0.605	0.255	1.440	0.256				
Location of the tumor: (0) central, (1) peripheral	1.817	0.764	4.319	0.177				
Type of resection: (0) anatomical, (1) nonanatomical	1.738	0.714	4.231	0.223				
Post-operative complications: (0) no, (1) yes	1.873	0.725	4.843	0.195				
Chest drain duration	0.888	0.681	1.159	0.383				
Hospital stay	1.013	0.787	1.306	0.917				
Mitoses	0.926	0.267	3.214	0.904				
Dimension of the tumor	0.994	0.942	1,.049	0.835				
T	0.042	0.000	21.761	0.320				
pStage								
Left lower lobe: (0) no, (1) yes	2.060	0.825	5.145	0.122				
Left upper lobe: (0) no, (1) yes	0.539	0.124	2.343	0.409				
Right lower lobe: (0) no, (1) yes	0.834	0.279	2.489	0.745				
Right upper lobe: (0) no, (1) yes	1.125	0.331	3.827	0.850				
Middle lobe: (0) no, (1) yes	0.751	0.272	2.070	0.580				

## Data Availability

The data underlying this article will be shared by the corresponding author upon reasonable request.

## Abbreviations

The following abbreviations are used in this manuscript:

TC	Typical carcinoid
OS	Overall survival
CCI	Charlson–Deyo comorbidity index
DFS	Disease-free survival
L-NETs	Lung neuroendocrine tumors
WHO	World Health Organization
AC	Atypical carcinoid
LCNEC	Large cell neuroendocrine carcinoma
SCLC	Small cell lung cancer
HPF	High-power fields
NCCN	National Comprehensive Cancer Network
ESMO	European Society for Medical Oncology
IASCL	International Association for the Study of Lung Cancer
CommNET	Commonwealth Neuroendocrine Tumour Research Collaboration
NANETS	North American Neuroendocrine Tumour Society
CT	Computed tomography
CDCC	Clavien–Dindo Complications Classification
CSS	Cancer specific survival
NSCLC	Non-small cell lung cancer
SEER	Surveillance, Epidemiology, and End Results
NET-WG	European Society of Thoracic Surgeon Neuroendocrine Tumours Working Group
